# Identification of novel inhibitors against hantaviruses through 2D fingerprinting and molecular modeling approaches

**DOI:** 10.3389/fimmu.2023.1113321

**Published:** 2023-02-08

**Authors:** Abdulrahman Alshammari

**Affiliations:** Department of Pharmacology and Toxicology, College of Pharmacy, King Saud University, Riyadh, Saudi Arabia

**Keywords:** hantaviruses, Gn-favipiravir, virtual screening, MD simulation, immunotherapeutics

## Abstract

With the immensely growing outbreaks of hantavirus with still no effective treatment available, there is an urgent need of exploring new computational approaches which will target potential virulent proteins that will eventually reduce its growth. In this study, an envelope glycoprotein, Gn, was targeted. The glycoproteins, which are the sole targets of neutralizing antibodies, drive virus entry *via* receptor-mediated endocytosis and endosomal membrane fusion. Inhibitors are herein proposed to negate its action mechanism. On the basis of the scaffolds of favipiravir, a FDA compound already used against hantavirus, a library was designed using a 2D fingerprinting approach. Upon molecular docking analysis, the top four docked compounds—(1) favipiravir (-4.5 kcal/mol), (2) N-hydroxy-3-oxo-3, 4-dihydropyrazine-2-carboxamide (-4.7 kcal/mol), (3) N, 5, 6-trimethyl-2-oxo-1H-pyrazine-3-carboxamide (-4.5 kcal/mol), and (4) 3-propyl-1H-pyrazin-2-one (-3.8)—were prioritized on the basis of the lowest binding energies score. Through molecular docking, the best-categorized compound was subjected to molecular dynamics simulation for a 100-ns time span. Molecular dynamics sheds light on each ligand behavior within the active site. Among the four complexes, only favipiravir and 6320122 compound were found to be stable inside the pocket. This is due to the presence of common rings, pyrazine and carboxamide ring, which make a significant interaction with active key residues Furthermore, the MMPB/GBSA binding free energy analysis calculated for all complexes supported the dynamics results by calculating the most stable values for favipiravir complex (-9.9933 and -8.6951 kcal/mol) and for 6320122 compound complex (-13.8675 and -9.3439 kcal/mol), which demonstrated that the selected compounds have a proper binding affinity with the targeted proteins. The hydrogen bond analysis similarly revealed a strong bonding interaction. The results yielded a strong interaction between the enzyme and the inhibitor throughout the simulation; thus, the inhibitor has the potential to become a lead compound and could be subjected to experimental evaluation to unveil their blockage ability.

## 1 Introduction

While the entire world is grappling with COVID-19, the outbreak of hantavirus also takes place (1). Hantaviruses belong to the family of *Bunyaviridae* which has over 300 species that are grouped into five subgroups, one of which is *Hantavirus* (2). Literature shows that other members of this family opted for the mode of transmission which is through arthropods, whereas hantavirus is transmitted from small mammals such as dogs, cat, sheep, and mainly rats. Until recently, it was believed that the main natural reservoirs for the human transmission of pathogenic hantaviruses were rodent bites, pee, saliva, or contact with rodent waste products (3). Hantavirus remains a serious public health threat. An estimated 200,000 persons per year have been exposed to contamination in recent years across the globe (4). Despite the fact that some nations have not yet recorded human hantavirus infection in their data, an increasing number of nations are reporting the virus’ emergence (5). Moreover, despite the fact that hantaviruses do not infect rats, they can nevertheless spread to people through insectivores, contaminated samples, and aerosolized rodent excrement as shown in **Supplementary Figure S1**. Hantavirus can cause potentially fatal illnesses in humans, such as hemorrhagic fever with renal syndrome and HPS, whereas others have not been linked to such illnesses (6). The surface of the virus is made of ribonucleic acid (RNA) (7). The three negative, single-stranded RNAs that make up the genome share the 3′ genome segment’s terminal sequence (8). The names of these three segments are large (L), medium (M), and small (S). It can also connect the L protein, the viral envelope RNA dependent polymerase, the nucleoprotein (N), and the glycoprotein (Gn) (3). The outer membrane of the hantavirus envelope displays a lattice of two glycoproteins, Gn and Gc, which orchestrate host cell recognition and entry. Glycoproteins help viruses in entering bodily cells, that is why Gn serves to be an important therapeutic or preventative target (4). Several drugs have been proposed against hantavirus, but to date no drugs are found against glycoproteins (5). Development and drug targeting identification is a multi-disciplinary, highly expensive, and time-consuming process. Scientific advancement during the past two decades have brought about several changes in the field of drug development and processing in such a way that advancement computational base approaches play a vital role and thus have enabled the identification of drug targets (9). On the bases of several computational approaches, structure and ligand base drug designing could speed up the drug development against several emerging viral strains as around the globe researchers are endeavoring to find out specific drugs that target identification against pathogenic viral strains to tackle them and reduce the infection rate (6). However, so far, no specific drug has been approved by the Food and Drug Administration against hantavirus. Drugs such as ribavirin, favipiravir, lactoferrin, and vandetanib were known to have been approved against ancient the species of hantavirus (7). In this study, we propose inhibitors that are similar to the FDA drug favipiravir that has shown experimentally effective results against Sin Nombre orthohantavirus and Andes virus *in vitro*. Moreover, this drug was approved for the newly emerging wave of influenza in 2014 in Japan and was also repurposed against COVID-19. This provokes us to use this drug in this study, with the sole purpose of generating analogs of favipiravir. Based on proteomics-based solution for the tackling of hantavirus and mainly depending on our understanding of the target proteins of hantavirus, a thorough step-wise conclusive approach is vital for understanding the role of target protein substitutions on the binding with target receptors; hence, the docking study is important in order to check the binding interaction between ligands and receptors. This analysis will also provide an insight to understand this structure-based interaction. Molecular dynamic simulation analysis is an *in silico* simulation approach mainly used to analyze the dynamic behavior of docked molecules and can analyze the binding stability of the docked molecules as well (8). Despite the fact that numerous studies have been conducted to identify and develop antiviral therapies and vaccines to prevent and treat hantavirus infections, there is currently no WHO- or FDA-approved vaccine or therapy available for patients. In the current study, 2D fingerprinting and molecular docking, followed by a detailed interaction analysis between the ligand and protein, were carried out. The shortlisted drug target candidates are tested in extensive molecular dynamics simulation to assess their real time behavior. The results were later further validated by binding free energy calculations and hydrogen bond analysis, which confirm the complex stability during the simulation run time. This study saves the cost of experimental laboratory resources as well as is time-saving in order to finally propose probable therapeutic substances which are possible potential targets to act as a good inhibitor against the target disease under consideration.

## 2 Research methodology

**Figure 1:**
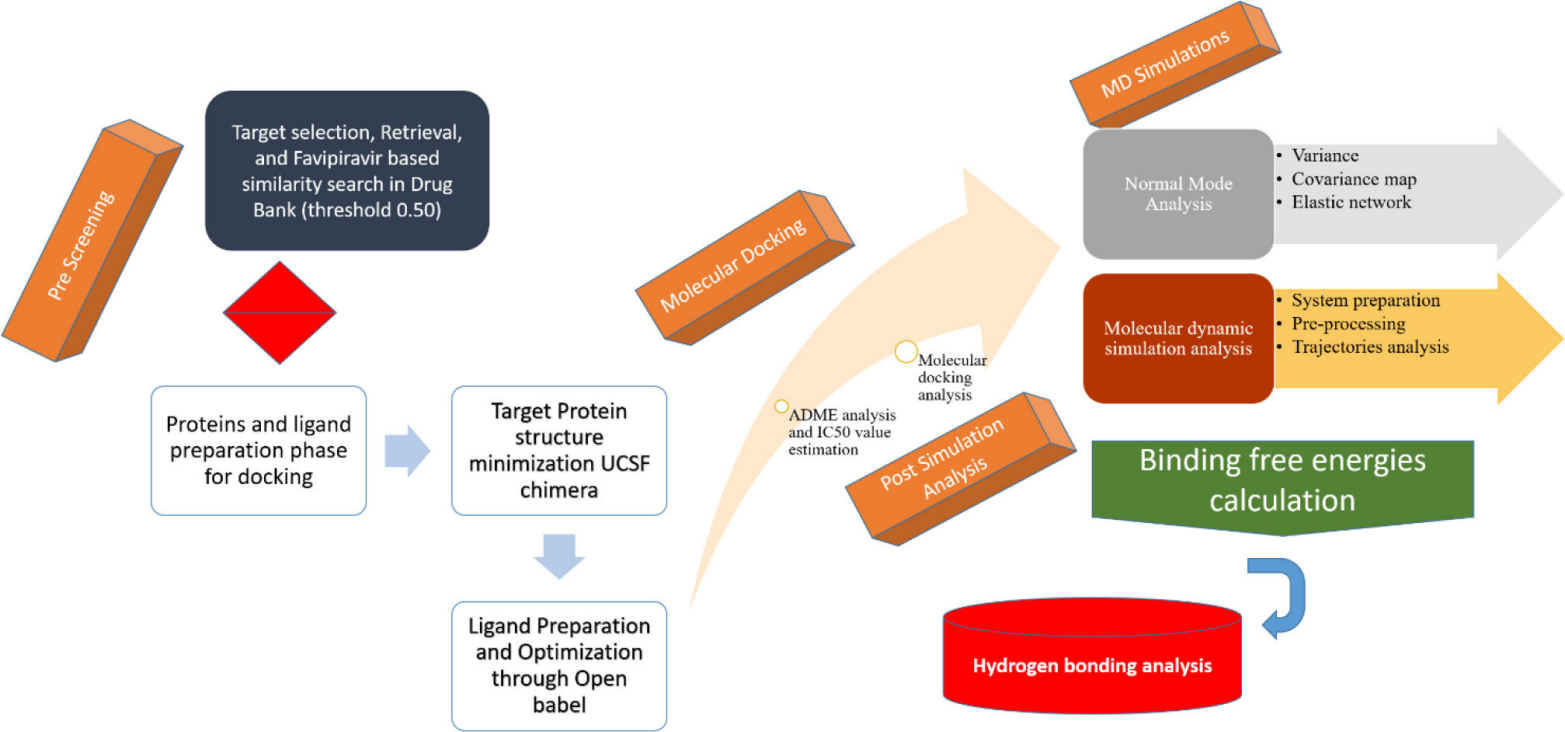
Overall flow diagram followed for targeting the Gn protein of hantavirus.

The schematic representation of the designed study is presented in **Figure 1**.

### 2.1 Structural modeling

The fasta sequence of Gn protein having PDB ID 6y6p was used to perform BLASTp to search the PDB for a suitable structure for the template. Based on query coverage, resolution, and sequence identity, the best templates were chosen (10). The 5OPG template was used, and it was tested using Swiss model (11) and Modeller (12). ERRAT, Verify3D, PROCHECK, and the RAMACHADRAN PLOT were among the quality evaluation tools used to assess the predicted models’ thermodynamic stability and quality (13).

### 2.2 Ligand-based similarity search

Favipiravir is an antiviral drug that can be used to treat other viral infections in addition to influenza. It serves as the parent molecule in the development of a library of inhibitors with similar scaffolds that target proteins. Based on the scaffolds in the PubChem repository, a 300-compound inhibitor library was created (14). To find favipiravir-like similar compounds, 70% Tanimoto coefficient was used as the evaluation criterion (15). All compounds were downloaded in.sdf format. Open Babel software was used to convert the compounds from 2D to 3D format, and ChemDraw software was used in the MM2 force field to optimize the compounds (16, 17). After that, the compounds and proteins were subjected to molecular docking.

### 2.3 Molecular docking approach

The prepared compounds and the best-modeled structure were proceeded for molecular docking. The protein was first minimized from UCSF Chimera software with the help of Tripos Force Field. The 750 steepest descent steps were used to remove highly adverse steric clashes. This was followed by 750 steps of conjugate gradient performed after finishing the steepest descent steps to further refine the structure (18). Preparation of protein and ligand is essential prior to molecular docking. AutoDockTools-1.5.6 was used to remove water atoms and the native ligand from the active site, add polar hydrogen atoms and charges, and convert the PDB files for the protein and ligand to PDBQT format (19, 20). The grid box was designed to target the Gn protein’s active site, with the center at X: 20.7 (Å), Y: 23.8 (Å), and Z: 65.5 (Å) and the grid box dimensions at X: 9 (Å), Y: 9 (Å), and Z: 9 (Å) (21). The compounds’ binding affinities were calculated and ranked based on their highest negative values of binding affinity, which corresponded to their best binding affinities. Chimera and Discovery Studio Client 2017 softwares were used to create 3D and 2D representations of protein–ligand complexes as mentioned in **Supplementary Figure S2** (22).

### 2.4 ADMET and toxicity analysis

To check the pharmacokinetics and toxicity parameters of favipiravir and the top-ranked docked analogs, the online webserver admetSAR was utilized (23). The admetSAR provided the complete profile of absorption, distribution, metabolism, excretion, and toxicity (ADMET) of favipiravir and the top selected analogs (24). The admetSAR is an open-source, user-friendly database that provides the ADMET properties of chemical entities by using their common names, SMILES, or structure similarity (25). It contains ADMET data profiles of more than 96,000 compounds with 45 different ADMET properties of FDA-approved, experimentally determined, and clinical trial compounds (26).

### 2.5 Molecular dynamic simulation

The top-ranked docked pose with minimum binding energy was further evaluated in molecular dynamic simulation (27). The inhibitor parameterization was done through general AMBER force field (28), while the receptor properties were calculated using the ff14SB force field (29). As shown in **Supplementary Figure S3**, the complex was integrated into a TIP3P water box. The system was neutralized by the addition of Na+ ions to it. Langevin dynamics was used to keep the system temperature stable after heating it to 300 K (NVT) for 20 ps. Restriction of 5 kcal/mol-A2 on carbon alpha atoms was allowed at a time step of 2 fs. During equilibration, the system was relaxed for 100 ps. For 100 ps, the system pressure was maintained using an NPT ensemble. Finally, a production run of 100 ns was completed at the rate of 2 fs. AMBER CPPTRAJ was used to examine the generated trajectories for structural parameters (30).

### 2.6 Hydrogen bond analysis

Hydrogen bonds are essential non-covalent interactions that occur when a hydrogen atom moves between the donor and acceptor atoms of an electronegative hydrogen bond (31). Hydrogen can absorb or give hydrogen when it is bound to oxygen, nitrogen, or fluorine. The hydrogen bond connections between receptor and ligand molecules were measured using the VMD plugin. A total of 5,000 frames of MD simulation were screened out to determine the number of hydrogen bonds created during the simulation (32).

### 2.7 MMPB/GBSA binding energy calculation

The MMPBSA.py module of AMBER18 (33) was used to calculate the solvation free energy and interaction energy for the receptor, ligand, and receptor–ligand complexes. The net binding free energy of the system was calculated as the average of the above-mentioned energies using the MM-PBSA method and its AMBER complement MM-GBSA to trace the difference between the bound and unbound states of a molecule’s solvated conformations. The following Eq (1). can be used to calculate the binding free energy mathematically:


$$\begin{array}{l} \Delta G\text{binding freeenergy}\\ =\Delta G\text{bind},\text{vaccum+}\Delta G\text{solv, complex}-(\Delta G\text{solv, ligand+}\Delta G\text{solv, receptor)(i)} \end{array}$$

ΔG solv = ΔG electrostatic, ∈ = 80 + ΔG electrostatic, ∈ = 1 + ΔG hydrophobic (ii)

ΔG vaccum = ΔE molecular, mechanics −T,ΔG normal mode analysis (iii)

The PB or GB equations were used to calculate the solvation energy for all system states, revealing the solvation state’s electrostatic contribution (28).

## 3 Results and discussion

### 3.1 Structural modeling

As the crystal structures of Gn reported in the PDB contain missing restudies, homology modeling was thus first performed *via* MODELER and SWISS-MODEL. Among them, the best generated model was chosen on the basis of physicochemical criteria and quality factors. Furthermore, the models were subjected towards structural analysis and verification servers. The analysis suggests that the best model was given by SWISS-MODEL server, with 89% residues, which is shown in **Supplementary Figure S4** and **Table 1**.

**Table 1:** Stereochemical property analysis modeled proteins.

Predicted 3D models	Errat	Verify3D	M.F.R.	A.A.R.	G.A.R.	Disallowed regions	Prosa web
Swiss model	93.0%	96%	89%	10.6%	0.0%	0.0%	-8.0
Modeller	34.4%	26	85%	11%	2%	1%	-3.1

M.F.R., mostly favored regions; A.A.R., additionally allowed regions; G.A.R., generally allowed regions.

### 3.2 Molecular docking

The field of structure-based drug design relies heavily on molecular docking to predict the binding mode and intermolecular framework of chemical interactions between small molecules and proteins. The active site residues (Pro29, Try117, Ser298, Gly299, Ile300, and Pro301) of Gn were found from literature and were opted for site-directed docking. Similar compounds obtained on the basis of favipiravir scaffolds were subjected for docking upon docking the top three compounds that were filtered out on the basis of binding energy value (kcal/mol) as listed in **Table 2**.

**Table 2:** Docking results of the top-hit compounds, with lowest binding energy score and interactive amino acid residues.

Compound structure	Compound name	Binding energy	Interacting residues
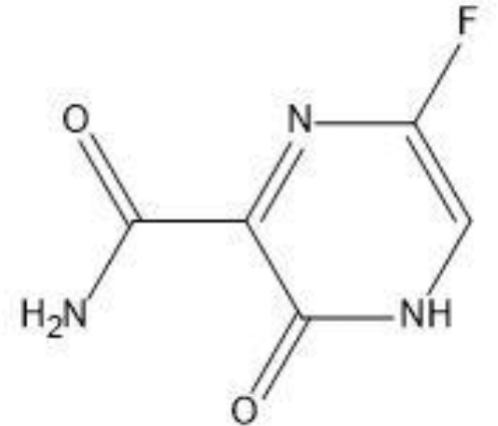	Favipiravir-5-fluoro-2-oxo-1H-pyrazine-3-carboxamide	-4.5	TRY297, SER (116–298), GLY299
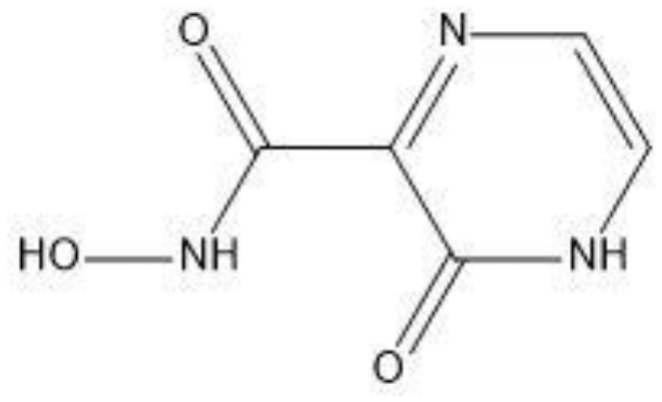	Structure2D_CID_6320122-(N-hydroxy-3-oxo-3,4-dihydropyrazine-2-carboxamide)	-4.7	ILE295, HIS259, PHE324
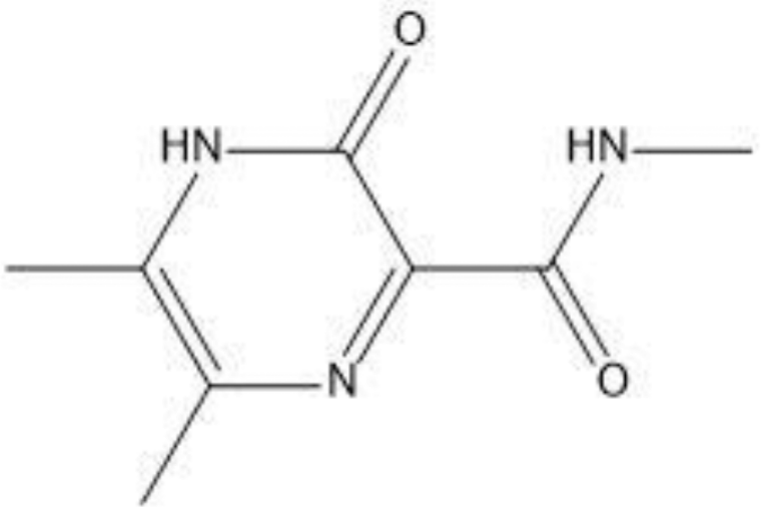	Structure2D_CID_308044-(N,5,6-trimethyl-2-oxo-1H-pyrazine-3-carboxamide)	-4	LEU294, LYS326
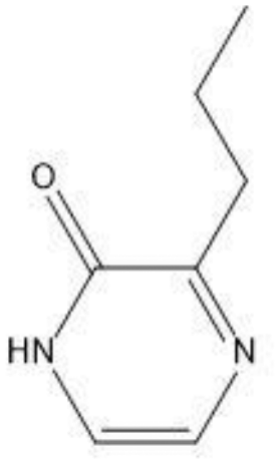	Structure2D_CID_12826353-(3-propyl-1H-pyrazin-2-one)	-3.8	HIS259

#### 3.2.1 Interaction analysis of Gn complexes

**Figure 2:**
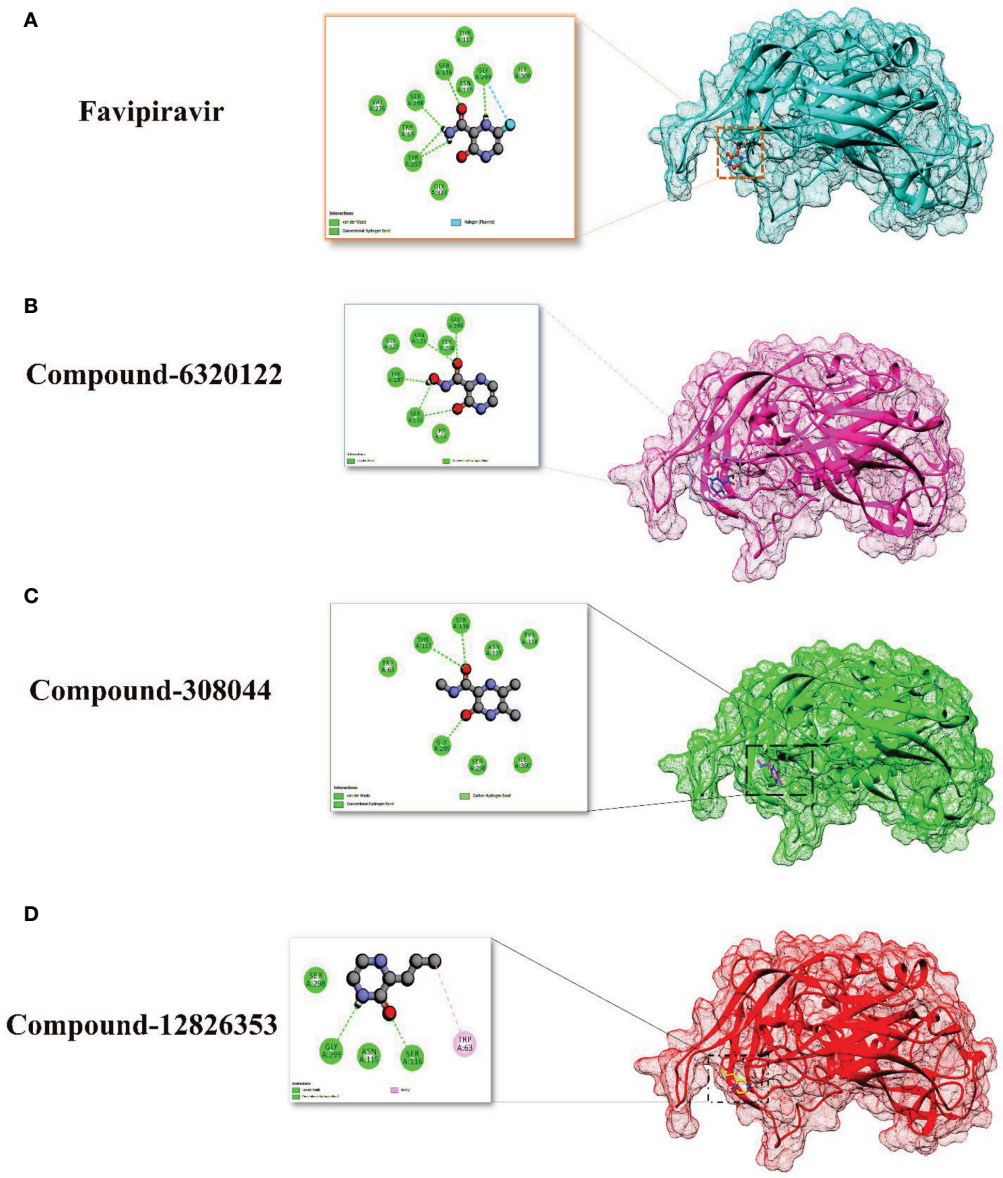
Depiction of the molecular interactions between the top selected candidates and Gn receptor **(A)** favipiravir, **(B)** 6320122, **(C)** 308044, and **(D)** 12826353.

For favipiravir compound, oxygen atoms of carboxamide scaffold form hydrogen bonds with SER298, TRY297, and SER116, whereas the dihydropyrazine ring N and F atom establishes a hydrogen and halogen bond with the GLY299 residue. Among the top compounds, compound 6320122 possesses a higher docking score with -4.7 kcal/mol. Similar to favipiravir, the oxygen atom of the carboxamide ring contributes to making a conventional hydrogen bond with SER298, TRY297, SER116, and GLY299 residues, whereas for both compound 308044 and compound 12826353, residues SER116 and GLY299 were found to be common, with the exception of THR116 and TRP63, respectively, as shown in **Figure 2**. Furthermore, these top-docked compounds were checked for ADMET properties, followed by advanced computational analysis to investigate the contribution of common residues, such as GLY299 and SER116, in defining the binding affinities at the active site and the role of ligand movement based on MD simulation results.

### 3.3 ADMET and toxicity

The admetSAR webserver was used to calculate the ADME and toxicity properties of favipiravir and its top-docked analogs (29). Different ADMET properties such as blood–brain barrier, human intestinal absorption, p-glycoprotein inhibition, CYP450 2C9 inhibitor, human ether-a-go-go-related gene inhibition, acute oral toxicity, and rat acute toxicity (LD_50_, mol/kg) were calculated. The calculated properties are summarized in **Table 3**. In the case of blood–brain barrier, all compounds showed a positive value with high probabilities. All top analogs selected along with favipiravir were found to be non-inhibitors of p-glycoprotein. The drugs should be a non-inhibitor of CYP450 2C9, as this enzyme is essential for the metabolism of drugs, so all compounds are non-inhibitors of CYP450 2C9 enzyme. Human intestinal absorption parameter is important in ADME, as it plays a key role in transporting drugs to the target. All compounds shared an acceptable range of human intestinal absorption profile and toxicity parameters, while Structure2D_CID_12826353 indicated the highest LD_50_ value in rat acute toxicity, thus demonstrating their non-toxicity. The dynamics of these compounds further reveal their favorability towards Gn proteins.

**Table 3:** Pharmacokinetic parameters of favipiravir and its top analogs.

Parameters	Favipiravir	Structure2D_CID_6320122	Structure2D_CID_308044	Structure2D_CID_12826353
Blood–brain barrier	+	+	+	+
	(0.81)	(0.64)	(0.5)	(0.80)
Human intestinal absorption	+	+	+	+
	(0.8.00)	(0.80)	(0.7)	(0.9)
P-glycoprotein inhibitor	Non-inhibitor	Non-inhibitor	Non-inhibitor	Non-inhibitor
	(0.7)	(0.86)	(0.9)	(0.90)
CYP450 2C9 inhibitor	Non-inhibitor	Non-inhibitor	Non-inhibitor	Inhibitor
	(0.80)	(0.68)	(0.90)	(0.80)
Human ether-a-go-go-related gene	Non-inhibitor	Non-inhibitor	Non-inhibitor	Non-inhibitor
	(0.94)	(0.82)	(0.9)	(0.90)
Acute oral toxicity	III	III	III	III
	(0.63)	(0.5)	(0.60)	(0.60)
Rat acute toxicity (LD_50_, mol/kg)	(2.27)	(2.1)	(2.20)	(2.3)

+, Positive.

### 3.4 Molecular dynamic simulation

**Figure 3:**
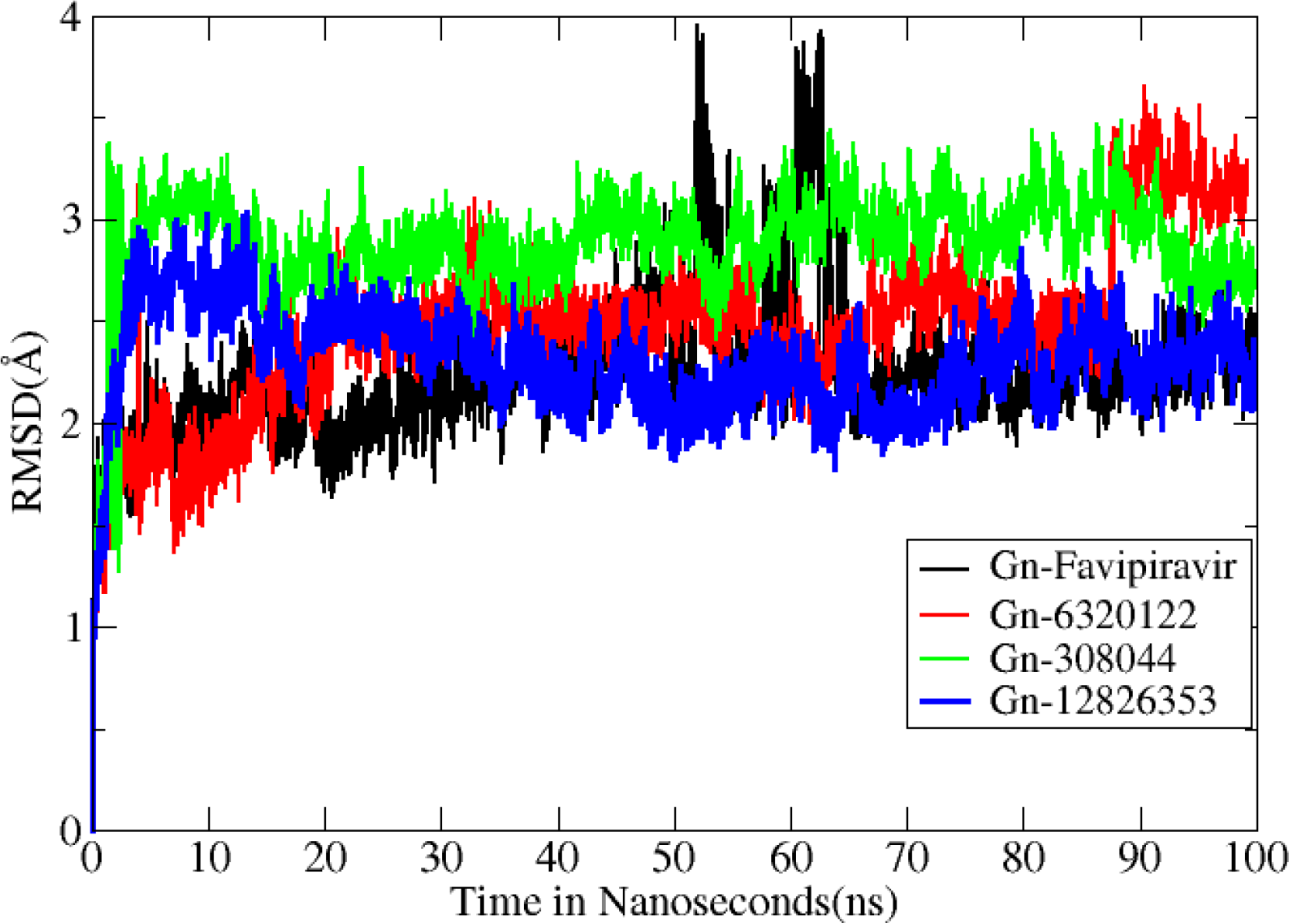
Root mean square deviation (Å) for all complexes. The Y axis denotes the root mean square deviation graph, and the X axis represents time in nanosecond (ns), which eventually highlight the changes that occurred at the protein structural organization per nanosecond.

**Figure 4:**
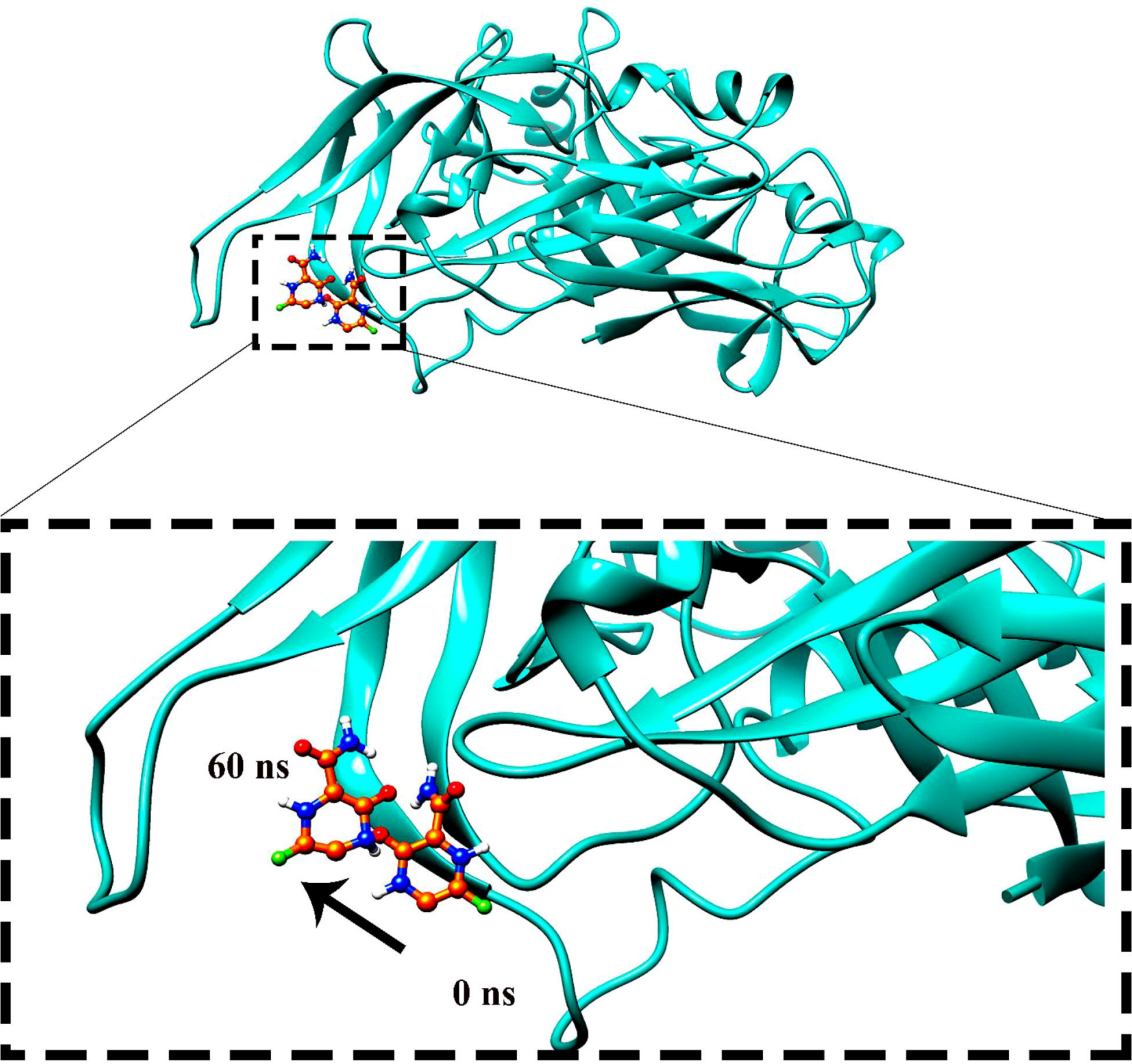
The movement of favipiravir compounds was noted in the binding site from 0 to 60 ns during the 100-ns time span.

**Figure 5:**
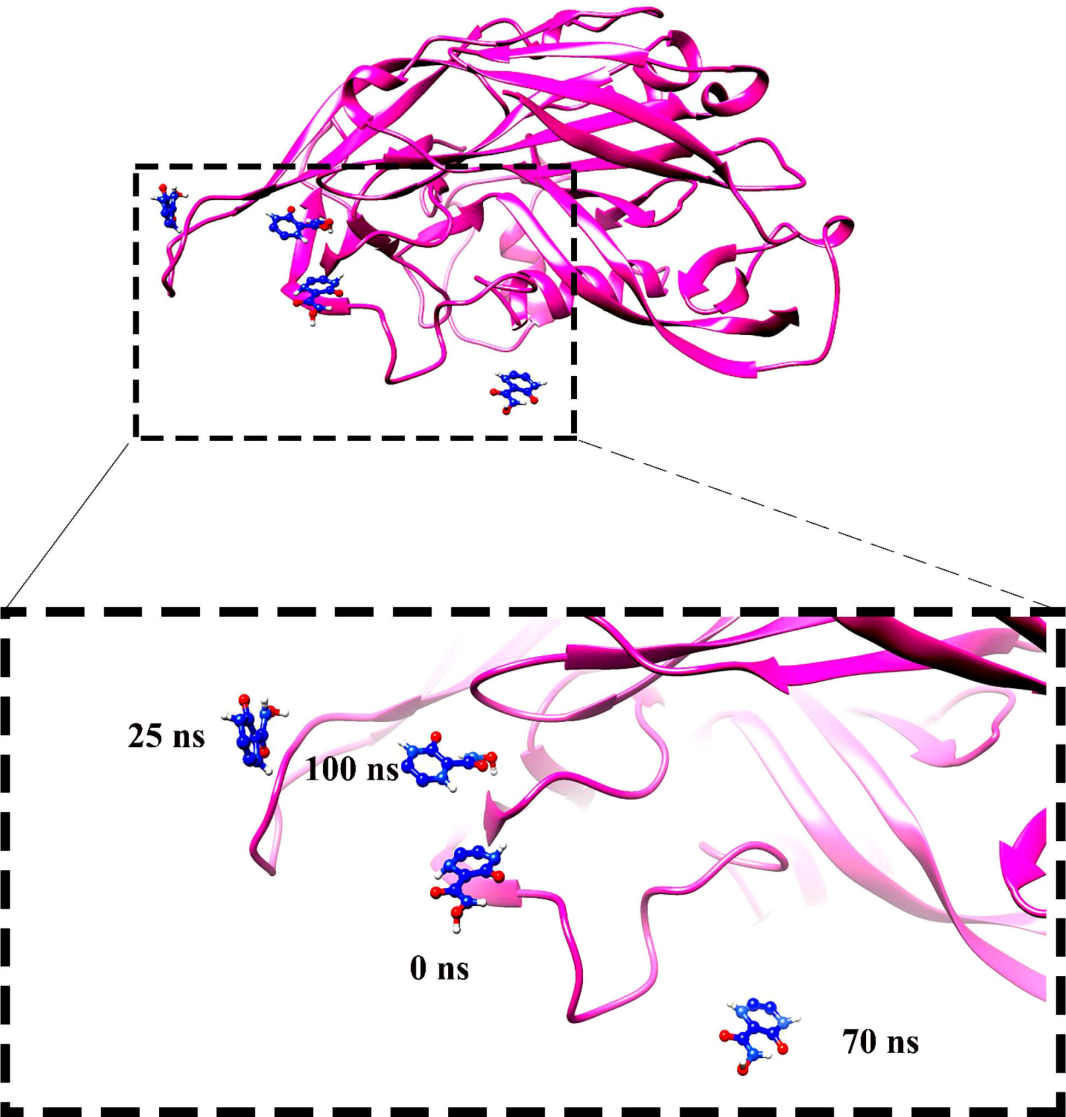
Compound 6320122 moved from the original docked position from the active site and swings in the anticlockwise direction and then re-positioned the ligand in the active site.

**Figure 6:**
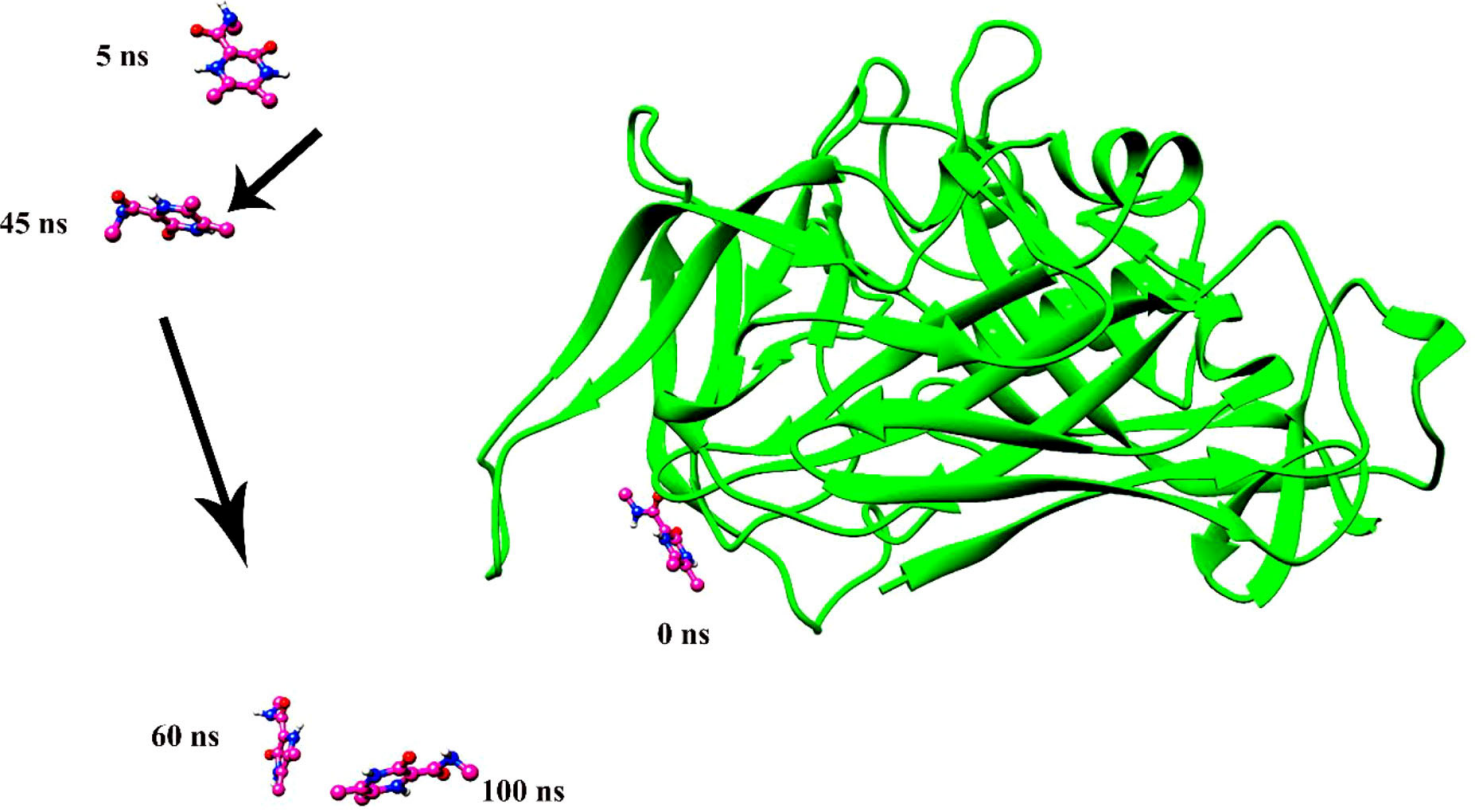
The instability of ligand 308044 was noted over a time span of 100 ns. The ligand leaves the pocket in the initial phase of the molecular dynamics simulation and continues to move in space.

**Figure 7:**
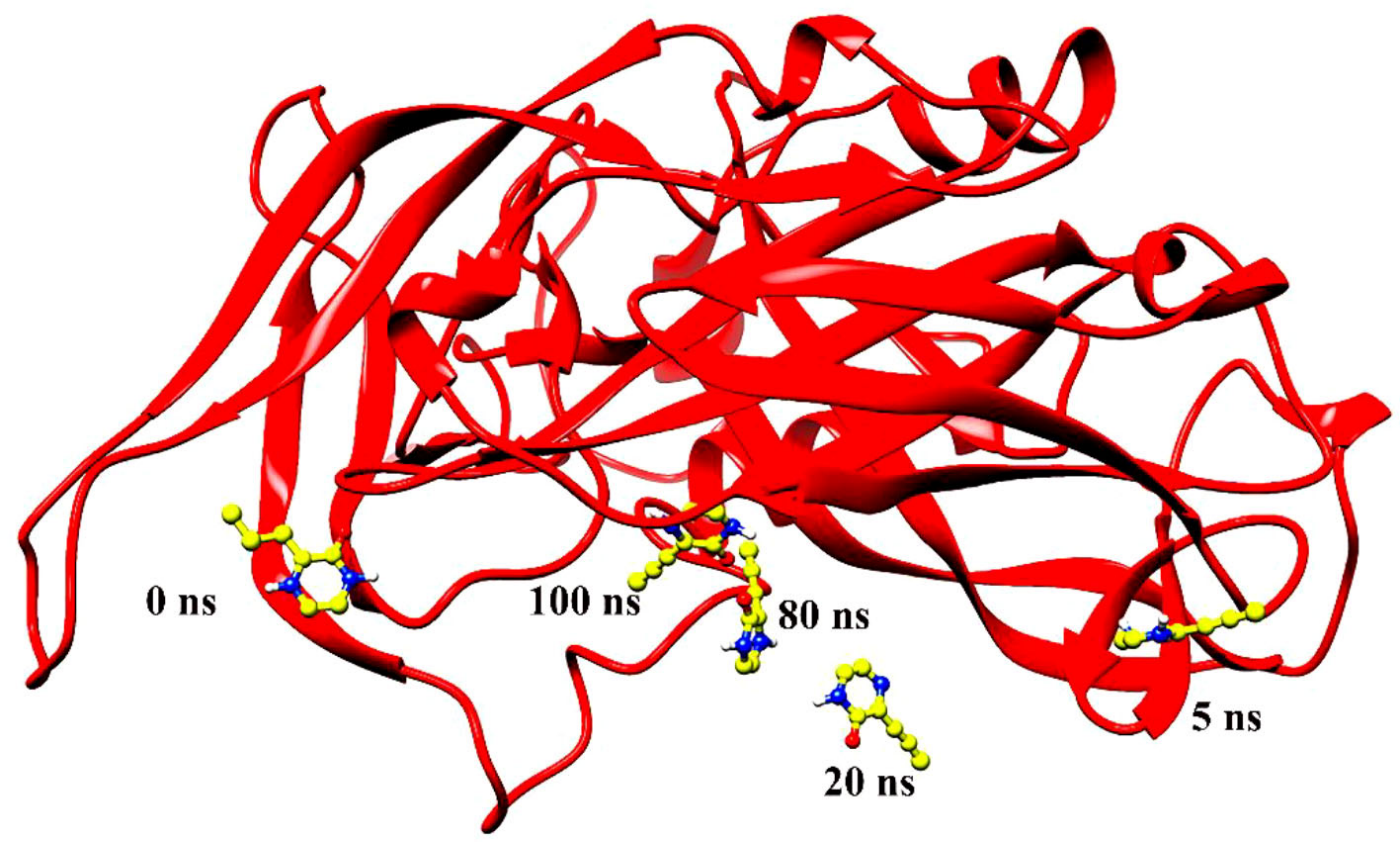
Ligand 12826353 left the pocket during simulation of 100 ns and remained swinging in the space around the protein.

Molecular dynamics simulation findings serve as an influential tool to observe the intimate conformational details taking place in biological systems. There were four protein systems subjected for MD simulations for 100 ns. The stability of complexes was monitored from RMSD. The RMSD values of Gn’s alpha carbon atoms in complex with favipiravir and its analogs were calculated using the original docked structure; these are Gn-favipiravir (maximum, 3.7Å and mean 2.24 Å), Gn-6320122 (maximum, 3.6Å and mean 2.46 Å), Gn-308044 (maximum, 3.4 Å and mean 2.28 Å), and Gn-12826353 (maximum, 3.04 Å and mean 2.30 Å). Upon inspection at the structural level, it was found that only few structural rearrangements take place. The major ligands move from the active site, as the size of the pocket is wider. In the case of Gn–favipiravir complex, a higher peak was noted in its RMSD plot. It was found that, at 60 ns, the ligand slightly shifted from its original docked position as shown in **Figure 3**. Similarly, in the case of favipiravir analogs, structural changes were monitored, which eventually shed light on the movement of the ligand in the pocket (**Figure 4**). In the case of Gn-6320122 complex, it was found that, at 25 ns, the ligand moved from the pocket and kept on moving outside the active site in an anticlockwise movement, but at nearly 100 ns, it entered the pocket again and remained there (**Figure 5**). Upon comparing with the dynamics of Gn-308044 and Gn-12826353 complex, it was found that both the chemical structures of ligands 308044 and 12826353 did not favor interacting with the binding pocket of Gn, that is why they were not retained in the pocket (**Figures 6**, **7**). In Gn-308044 complex, at the start of simulation, the ligand moved away from the pocket and, at 50 ns, remained at the far distance from the pocket. At nearly 100 ns, the ligand tried to be in close vicinity to the binding site but still did not reenter in the pocket. Such similar trend and behavior were also observed for the Gn-12826353 complex. In order to further validate the molecular dynamic simulation results, the complexes were further subjected to free binding energy analysis.

### 3.5 MMPB/GBSA binding free energy calculation

The top compounds’ MMPB/GBSA-based binding energy and molecular interactions were calculated. For energy calculation, hundreds of snapshots of the trajectory analysis were evaluated. Convergence of average values determined by MMPB/GBSA is required to obtain reliable results from absolute binding free energies. Upon validating the molecular dynamic simulation results, it was found from the results also that Gn-favipiravir and Gn-6320122 form a stable complex compared with the other complexes. In stabilizing favipiravir, both Van der Walls and electrostatic interactions play a key role in stabilizing it in the Gn active site, whereas for the Gn-6320122 complex, electrostatic interactions were more dominant. As shown in **Table 4**, the combined average values of gas phase binding free energy using both MMPBSA and MMGBSA methods were found to be significant in contributing to complex stabilization.

**Table 4:** Binding energy calculation for favipiravir and its analogs.

Energy components	Gn-favipiravir		Gn-6320122		Gn-308044		Gn-12826353	
	MMPBSA	MMGBSA	MMPBSA	MMGBSA	MMPBSA	MMGBSA	MMPBSA	MMGBSA
EEL	-9.61	-9.61	-168.17	-168.17	-572.07	-572.07	-298.47	-298.47
VDWAALS	-9.47	-9.47	-4.78	-4.78	-6.80	-6.80	-12.21	-12.21
DELTA G gas	-19.09	-19.09	-172.96	-172.96	-578.87	-578.87	-510.68	-510.68
DELTAG solv	9.10	10.39	159.09	163.61	566.47	572.48	488.04	494.33
Total energy	-9.99	-8.69	-13.86	-9.34	-12.39	-6.38	-7.64	-6.35

### 3.6 Per residue energy decomposition

**Figure 8:**
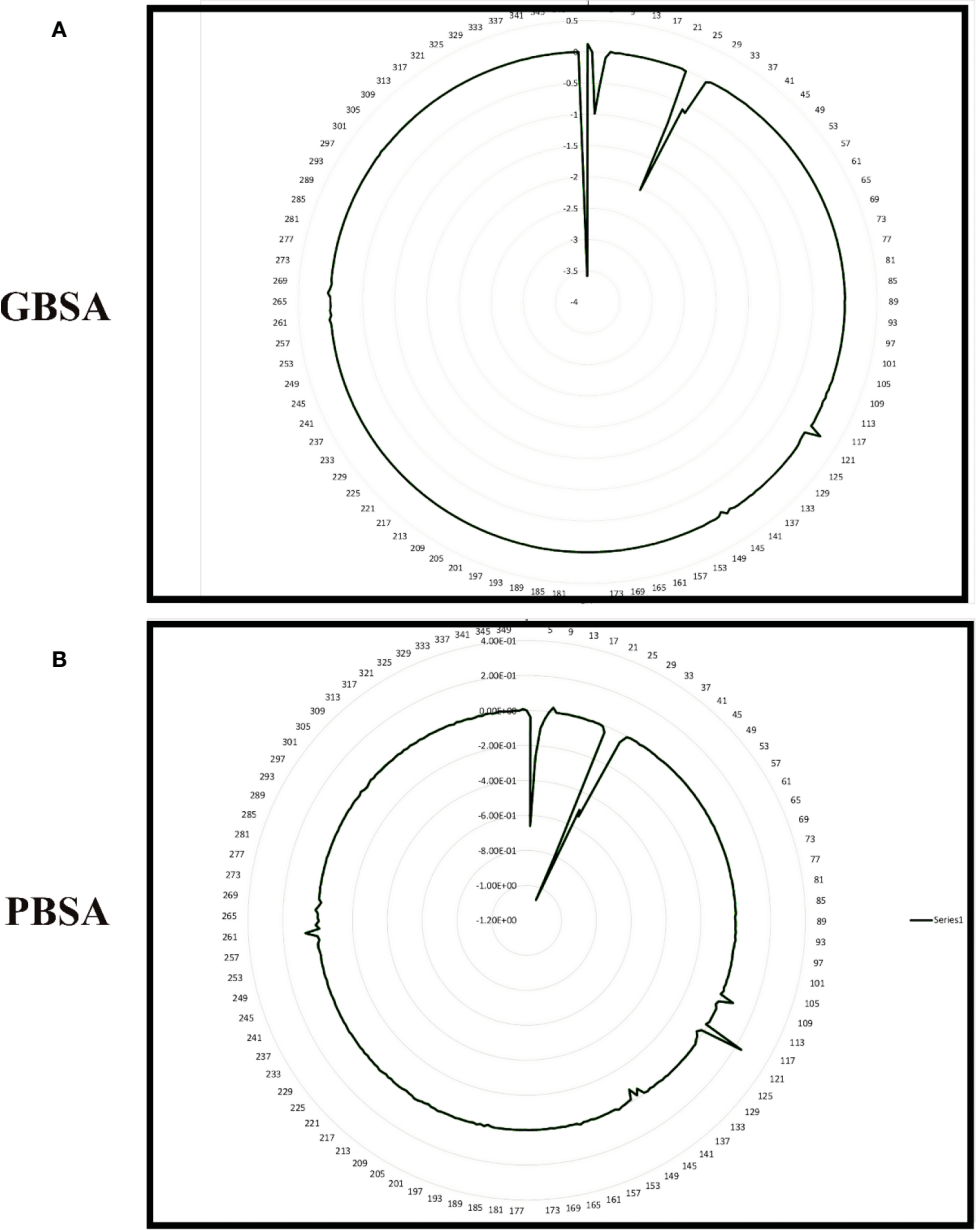
Per residue free binding energy calculation representation for Gn-favipiravir based on **(A)** MMGBSA and **(B)** MMPBSA.

**Figure 9:**
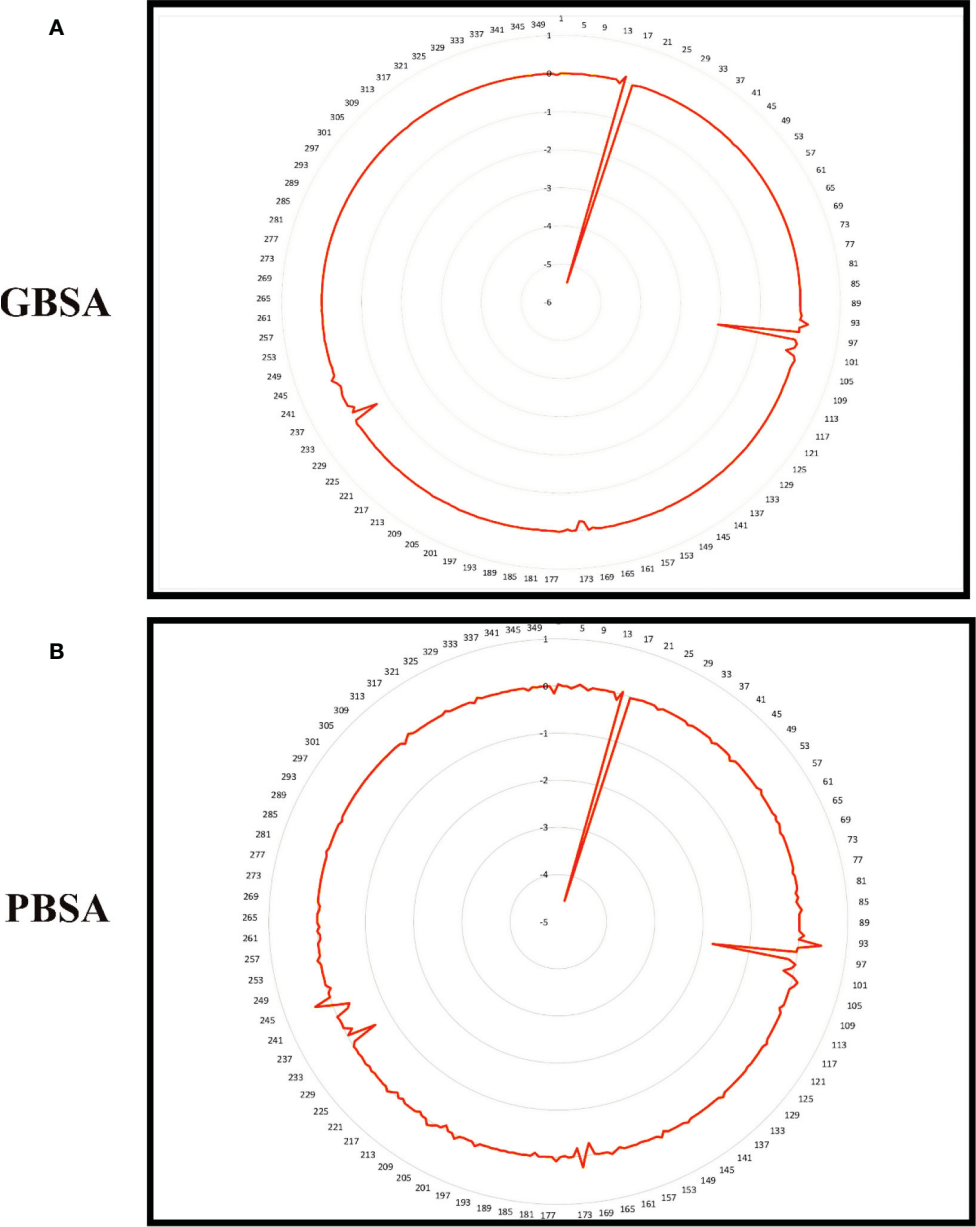
Per residue free binding energy calculation representation for Gn-6320122 based on **(A)** MMGBSA and **(B)** MMPBSA.

To improve our knowledge of how residues contribute energy to complex stabilization, per residue energy is calculated. All residues are classified as hotspot amino acids because they contribute less than -1 kcal/mol energy and play an important role in stabilizing the docked complex system. The GB analysis residues with binding energy less than 1 kcal/mol for Gn-favipiravir include ALA 278 (-7), ASN279 (-5), ALA286 (-5), ILE294 (-7), and TYR296 (-7), whereas in the case where PB residues were involved, these were SER287 (-2), ALA286 (-5), ALA295 (-7), and TRY296 (-0.002). In terms of residues that have a binding energy less than 1 kcal/mol in GB for Gn-6320122, these include GLU17 (-6), GLU97 (-2), and ARG101 (-0.22), whereas in the case where PB residues were involved, these were GLU17 (-4.5), GLU97 (-1.7), and ARG101 (-0.2). These residues lie in close proximity to the active site of Gn and considerably have low energy value, thus highlighting the importance of these residues presented in **Figures 8**, **9**.

### 3.7 Hydrogen bond

**Figure 10:**
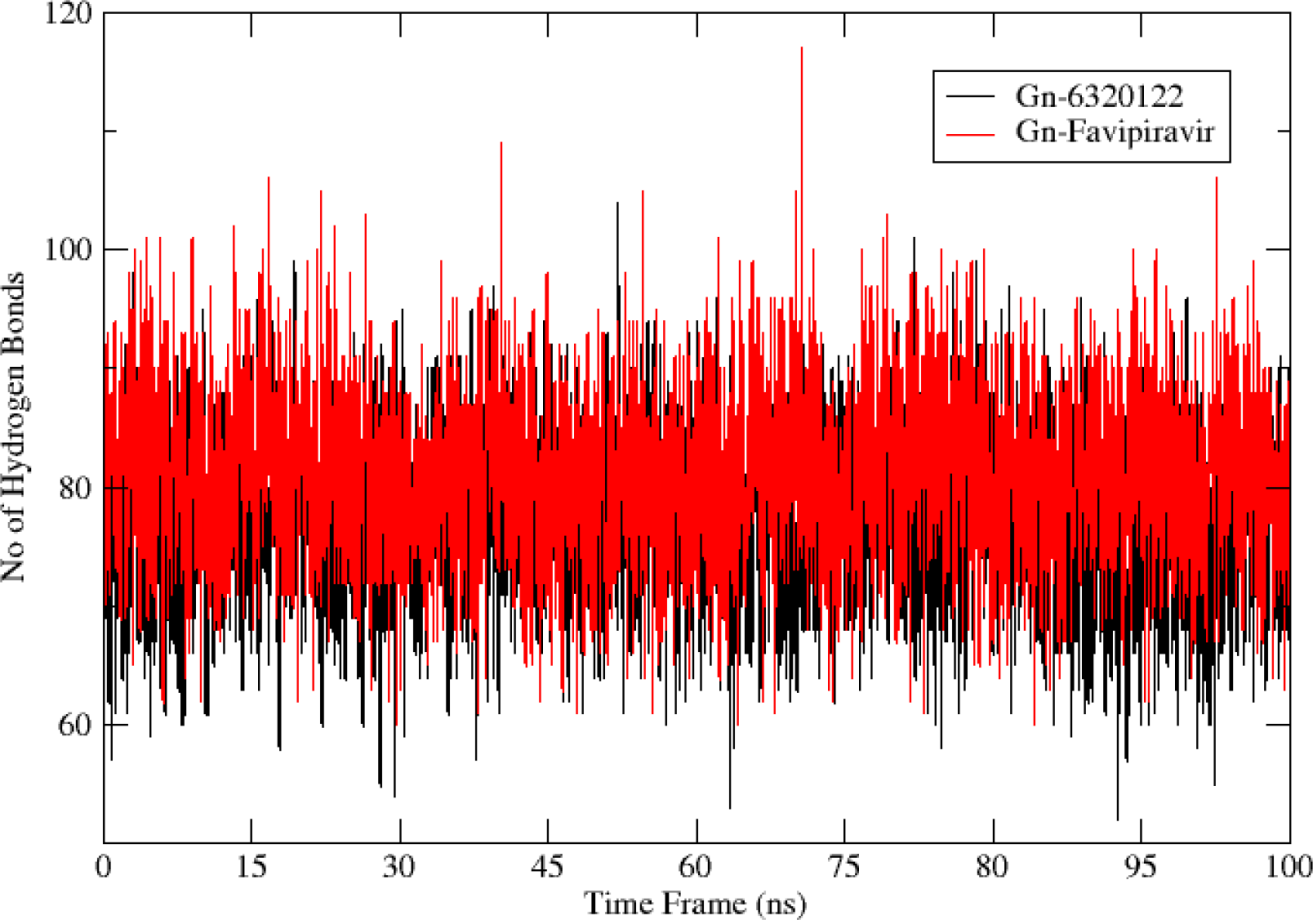
Hydrogen bond analysis for top complexes obtained from molecular dynamics simulation. The red color bonds depict the hydrogen bond frequency of Gn-favipiravir, while the bonds in black are of Gn-6320122.

The greater the number of hydrogen bond interactions, the greater is the strength of the ligand–protein interaction. To map the number of hydrogen bonds for Gn-favipiravir and Gn-6320122, hydrogen bond analysis was performed. The hydrogen bond analysis reveals that both ligands made hydrogen bonds with their respective protein residues within the range of 0–100, which is a good range for a stable interaction as shown in **Figure 10**.

## 4 Conclusion

In this study, 2D fingerprinting and structure-based virtual screening were performed using an FDA drug—favipiravir—to find potential candidates against Gn protein which is an important target in combating the hantavirus. Upon molecular docking, three top analogs of favipiravir were found. These potential candidates were further subjected towards extensive molecular dynamic simulation to investigate the dynamics of these candidates in a real system. The dynamics highlighted two best compounds, favipiravir and 6320122, that were found to be stable. The dynamics revealed that the presence of pyrazine and carboxamide ring in their structure allows them to vastly bind to the active site residues. Hence, the outcome of this study not only suggested favipiravir and 6320122 compounds as best potential inhibitors that must undergo *in vitro*, *in vivo*, and clinical trial phases in the future but also highlighted the importance of these rings (pyrazine and carboxamide) in compounds. This directs the future researchers in the domain of drug designing to have primary focuses on pyrazine and carboxamide ring chemical scaffolds in designing valuable inhibitors against the hantavirus.

## Data availability statement

The original contributions presented in the study are included in the article/**Supplementary Material**. Further inquiries can be directed to the corresponding author.

## Author contributions

The author confirms being the sole contributor of this work and has approved it for publication.
