# Supplementary material for: Identification of novel inhibitors against hantaviruses through 2D fingerprinting and molecular modeling approaches
Source: Front Immunol. 2023 Feb 8;14:1113321. doi: 10.3389/fimmu.2023.1113321 (PMC9944044; doi:10.3389/fimmu.2023.1113321)
Supplement: Supplementary file 1 [file DataSheet_1.docx]

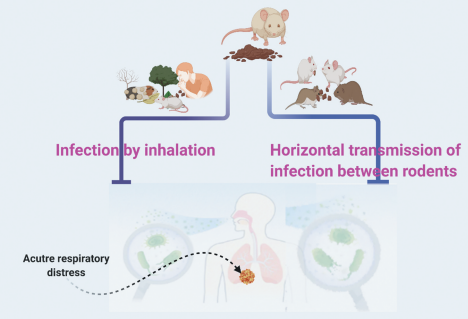


**S-Figure 1.** Transmission way of Hantavirus from rodent to human (Zonosis)

**
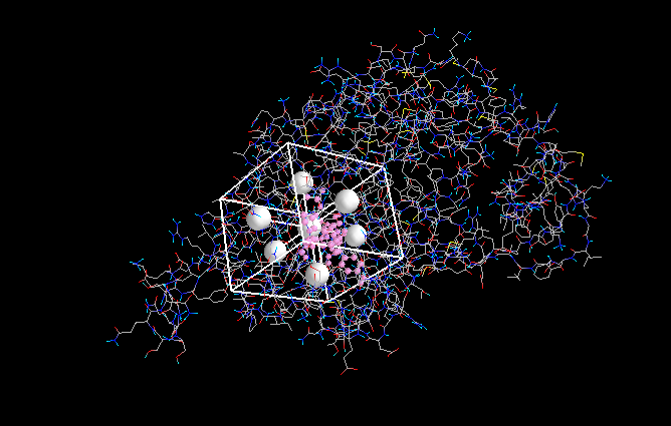
**

**S-Figure 2.** Depiction of Docking grid around active site for targeting compounds


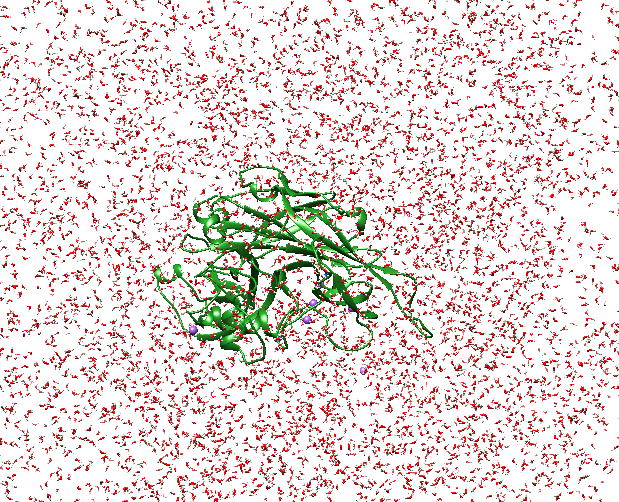


**S-Figure 3**. Integration of docked complexes into a TIP3P water box.

**
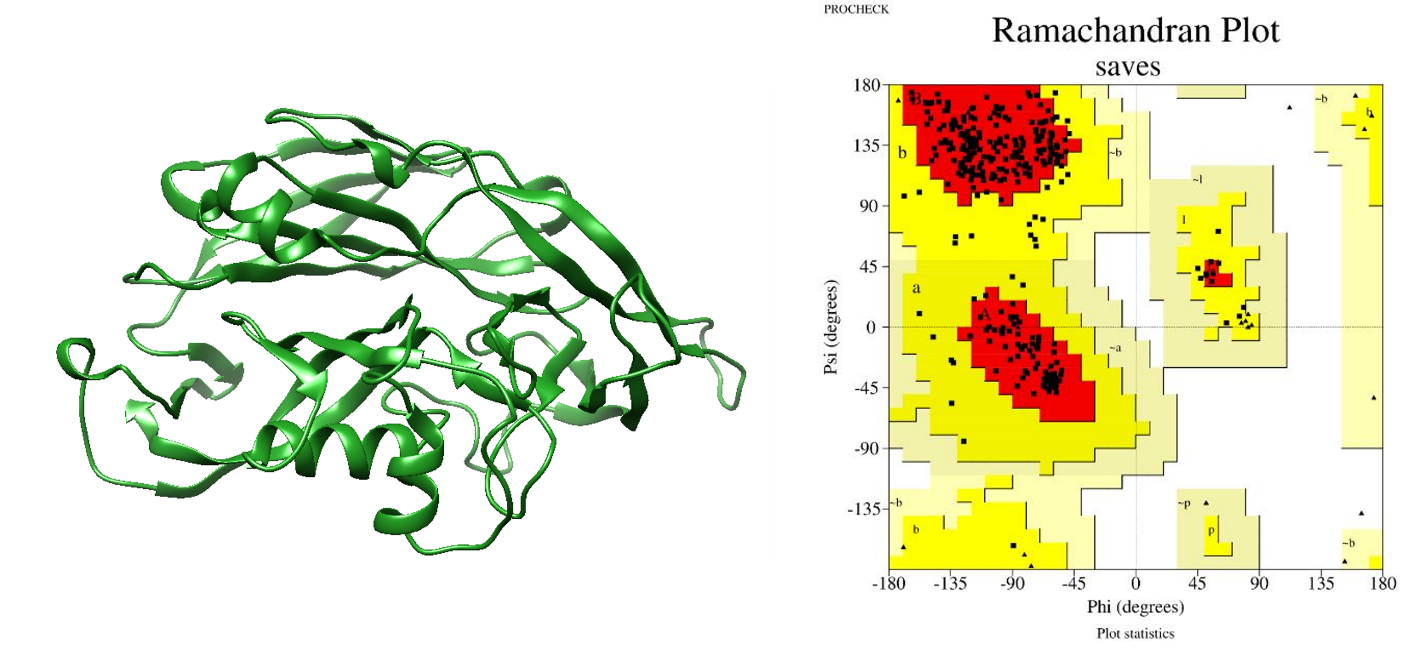
Figure S-Figure 4.** Representing modelled protein structure and its Ramachandran plot
